# Manganese-Loaded pH-Responsive DNA Hydrogels Enable Tg-Guided Thyroid Tumor Targeted Magnetic Resonance Imaging

**DOI:** 10.1021/acsami.4c19676

**Published:** 2025-02-25

**Authors:** Qingyi Hu, Anwen Ren, Ximeng Zhang, Zimei Tang, Rong Wang, Dong-Yuan Wang, Tao Huang, Jie Liu, Jie Ming

**Affiliations:** † Department of Breast and Thyroid Surgery, Union Hospital, Tongji Medical College, Huazhong University of Science and Technology, 430022, Wuhan, China; ‡ Department of Pharmacy, Union Hospital, Tongji Medical College, Huazhong University of Science and Technology, 430022, Wuhan, China; § Department of Radiology, Union Hospital, Tongji Medical College, Huazhong University of Science and Technology, 430022, Wuhan, China; 4 Hubei Provincial Clinical Research Center for Precision Radiology & Interventional Medicine, 430022 Wuhan, China; 5 Hubei Key Laboratory of Molecular Imaging, 430022 Wuhan, China

**Keywords:** DNA hydrogel, MR imaging, contrast agent, aptamer, thyroid cancer

## Abstract

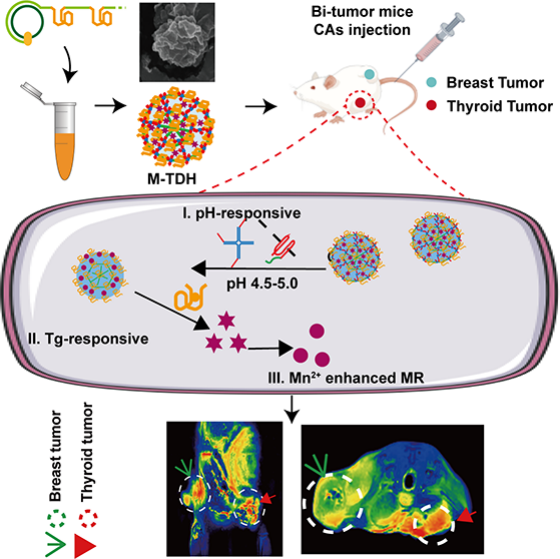

The diagnosis of metastatic and recurrent occult thyroid cancer presents a significant challenge. This study introduces a DNA-Mn hydrogel (M-TDH) that specifically targets thyroglobulin (Tg). This nanogel is loaded with paramagnetic Mn^2+^ for facilitating magnetic resonance (MR) imaging. As a cofactor of DNA polymerase, Mn^2+^ promotes the extension of long-strand DNA and forms Mn_2_PPi nuclei with PPi^4–^ in the system. The synthesis of M-TDH is achieved through Mn_2_PPi nucleation and growth with long-strand DNA acting as the structural framework. The X-scaffold functions as a junction point, thereby enhancing structural stability. The Tg aptamer sequence is incorporated into M-TDH, ensuring specific targeting of thyroid cancer cells. Furthermore, M-TDH demonstrates an extended residence time at the thyroid tumor site, thus increasing the duration of enhanced MR imaging. Overall, this study introduces an aptamer-based, thyroid tumor-targeted DNA nanogel for MR imaging diagnostic applications, with the potential to advance a multifunctional magnetic nanosystem toward clinical application.

## 1 Introduction

The incidence of thyroid cancer has increased globally over the past three decades.^1,2^ Although the 10-year survival rate for adult papillary thyroid cancer (PTC) patients is over 90%,^3^ there are 5–20% of patients experiencing local or regional recurrence and 10–15% of patients developing distant metastases, significantly compromising their survival.^4,5^ Magnetic resonance imaging (MRI) offers multidirectional imaging, high-resolution soft tissue imaging, no radiation exposure, and multiparameter imaging advantages.^6−9^ Contrast agents (CAs) used in MRI further enhance diagnostic capabilities. However, current imaging methods lack thyroid specificity, hindering the detection of occult thyroid tumor.

Nanogels are three-dimensional (3D) platforms composed of amphiphilic polymer chains formed by physical or chemical cross-linking.^10,11^ DNA nanogels exhibit excellent biocompatibility, versatility, and biostability, rendering them highly promising for biomedical applications.^12−14^ Aptamers are functional oligonucleotide sequences that target specific molecules,^15,16^ thereby facilitating directional transport of DNA materials. Additionally, DNA nanogels can load various imaging agents or drugs, thus enabling the diagnosis and treatment of tumors. For example, the rolling circle amplification (RCA) process using Mn^2+^ as a φ29 DNA polymerase cofactor has been explored to prepare DNA-Mn microparticles for enhanced MRI.^17^ Another study reported a DNA-Mn hybrid nanoflower (DMNF) used as an MRI CA for tumor imaging.^18^ Due to their excellent paramagnetic properties, Mn-based nanogels have been employed as CAs for MRI in many studies.^19,20^ However, a need remains for the development of new multifunctional nanogels to enhance the enrichment of Mn CAs at target sites and reduce the residual presence in other major organs.

This study aims to develop DNA nanogels targeting thyroid-originated cancers, loaded with Mn^2+^, for T1-weighted MRI. These nanogels are synthesized through an RCA reaction, scaffold assembly, and Mn-induced biomineralization. Upon entering the tumor tissue, Mn^2+^ is released via stepwise cleavage in response to the acidic pH and thyroglobulin (Tg), enabling MR enhancement imaging at specific sites. The key innovations of this system are the following: (1) In an acidic environment, I-linkers in the scaffold form triplex-DNA structures through Hoogsteen base pairing, disrupting the stability of nanogels and releasing Mn^2+^. (2) Aptamer targeting and recognition of Tg promote aggregation in thyroid-originated cancers and facilitate further cleavage of nanogels at the target site. (3) The larger particle size prolongs the blood circulation time and increases enrichment at target sites.

## 2 Materials and Methods

### 2.1 Materials and Reagents

Manganese chloride (MnCl_2_, AR, >99%), dimethyl sulfoxide (DMSO, cell culture grade, >99.5%), 4′,6-diamidino-2-phenylindole (DAPI) solution (>90%), and Dio (10 mg) were purchased from Solarbio Science & Technology Co. Ltd. (Beijing, China). Roswell Park Memorial Institute (RPMI) 1640 medium, Dulbecco’s modified Eagle medium (DMEM), and fetal bovine serum (FBS) were purchased from Gibco (Darmstadt, Germany). The cell counting kit-8 (CCK-8) was purchased from Selleck (Shanghai, China). The Annexin V-FITC/PI apoptosis detection kit was purchased from Vazyme (Jiangsu, China). Gadoteric acid meglumine salt Injection (15 mL:5.654 g Gd-DPTA) was purchased from Hengrui Pharmaceuticals Co., Ltd. (Jiangsu, China). Anti-Tg was purchased from Proteintech Group, Inc. (Rosemont, USA). φ29 DNA polymerase, T4 DNA ligase, dNTPs, DNA size markers, and 4S green nucleic acid stain were purchased from Sangon Biotech Co. Ltd. (Shanghai, China). All related DNA sequences were synthesized and purified by Sangon. A 10× Thermopol reaction buffer was purchased from New England Biolabs Beijing Co., Ltd. (Beijing, China). The BeyoChIP assay kit was purchased from Beyotime Biotechnology (Shanghai, China) for DNA–protein interaction detection.

### 2.2 Instruments and Characterization

The absorbance value at 260 nm was measured by a NanoDrop 2000c photometer (Thermo Scientific, America) to evaluate the DNA content in the RCA mixture. Field emission scanning electron microscopy (Noca NanoSEM 450 FEI Company, Netherlands) was used to investigate the morphology. Nanoparticle size and zeta potential analyzer (DLS, Malvern Zetasizer Nano ZS90) was used to analyze the diameter size of the nanoparticle. X-ray photoelectron spectroscopy (XPS, Thermo Scientific K-Alpha) was used to analyze the elementary components of M-TDH. The Mn content in DMNF was determined by inductively coupled plasma mass spectrometry (ICP-MS Agilent 7700, USA). The fluorescent images were obtained by an inverted fluorescent microscope (DMi8, Leica, Germany) and confocal laser scanning microscopy (CLSM, nikon A1 HD25/A1R, Japan). A chemiluminescence gel imaging system (ChemiDoc XRS+, Bio-Red, USA) was used to scan nucleic acids in agarose gel. An automatic microplate reader (Multiskan FC, Thermofisher, USA) was used to acquire the absorbance and fluorescence spectra. The quantification of cellular uptake was obtained by full spectrum analysis flow cytometry (ID7000 Sony, Japan). The MR imaging was performed on a 3.0 T magnetic resonance imaging system (Ingenia CX, Fhilips, The Netherlands).

### 2.3 Experimental Section

#### Synthesis of the Circular DNA Template

A 100 ng amount of phosphorylated padlock DNA and 300 ng of LT were mixed with a ratio of 1:3 in 1× T4 ligase buffer solution. The mixture was heated at 95 °C for 2 min and 55 °C for 5 min and gradually cooled to 20 °C using a PCR thermal cycler. After annealing, 10 U of T4 DNA ligase was added, and the reaction solution was incubated at 16 °C for 8 h. Then the solution was incubated at 4 °C overnight. The solution was heated to 65 °C for 10 min to inactivate the T4 DNA ligase.

#### Synthesis of the X-Scaffold

X1–4 strands (120 μM) and 150 μM I-linker were added into 1× Thermopol buffer to a final volume of 10 μL. The mixture was heated at 95 °C for 5 min and 55 °C for 5 min and gradually cooled to 25 °C at the rate of 1 °C/min using a PCR thermal cycler. The X-scaffold was stored at 4 °C.

#### M-TDH Preparation

The circular DNA template (0.5 μM), dNTPs (1.25 mM), RCA-primer (400 ng), and 10 U φ29 DNA polymerase were added sequentially into 1× φ29 DNA buffer containing 8 mM MnCl_2_ (pH 7.4) to a final volume of 40 μL. After the RCA reaction was kept at 30 °C for different times, 10 μL of X-scaffold and dNTPs (0.75 mM) were added into the RCA mixture with a final volume of 50 μL to continue RCA and perform a biomineralization reaction at 30 °C for another 2 h. After heating at 65 °C for 10 min to inactivate the φ29 DNA polymerase, the reaction was terminated. The M-TDH was washed three times with DEPC water and centrifuged at 12 000 rpm for 5 min. The M-TDH was dispersed in PBS or saline with a concentration of 6 mg/mL and stored at 4 °C. The RCA products were verified by 2% agarose gel at 120 V for 1 h with Tris-borate-EDTA (TBE, pH 8.0) and stained with green nucleic acid stain (1:10 000). The gel was scanned using a gel imaging analysis system. The specific Tg-aptamer sequence in TDH was detected by 4 pairs of primers and an RT-qPCR procedure with 4 pairs of primers. DNA sequences used in the synthesis of M-TDH and primers are shown in Table S1 and Table S2.

#### Determination of DNA Content in M-TDH

The total Mn content was quantified by ICP-MS. According to ref (18), the P content in the Mn_2_PPi was determined based on the molecular formula and Mn content, and the rest of P was contributed by DNA. As the P content in DNA was about 9.2%, the DNA content in M-TDH was then determined to be 19.3%, while the Mn content in M-TDH was calculated to be 31.1%. The content of DNA in the Mn_2_PPi framework was calculated to be 0.24 g of DNA per g of Mn_2_PPi. The DNA content in RCA products was determined by a Nanodrop photometer. The RCA reaction mixture was diluted to 1/10 with DEPC water and heated to 95 °C. The absorbance value at 260 nm was measured by a Nanodrop photometer. The DNA concentration (*C*_DNA_) was calculated as follows: *C*_DNA_ = OD_260_ × 40 ng/μL.

#### Cell Culture and Cellular Uptake

The human thyroid cancer cell lines (TPC-1 and K1) and breast cancer cell lines (MDA-MB-231 and MCF-7) were incubated in DMEM with 10% FBS. The human normal thyroid cells (Nthy ori-3.1) were cultured in RPIM-1640 with 10% FBS, and human breast epithelial cells (MCF-10A) were cultured in specific medium (Pricella, CM-0525). All cells were cultured at 37 °C under a humidified atmosphere of 5% CO_2_. Those cells were digested with trypsin and resuspended in medium. As for fluorescent microscope observation, the cells were seeded in a six-well plate with a density of 10 000 cells per well. After incubating with 50 μL of reagents containing Cy5 for a specific time, the cells were fixed in 4% paraformaldehyde, stained with DAPI and Dio, and then observed by fluorescent microscope. The DAPI-stained nucleus was shown in blue, Dio-stained membranes in green, and Cy5 in red. For flow cytometry, the cells were seeded in six-well culture plates overnight and incubated with 50 μL of reagents for specific times. Then cells were digested with trypsin without EDTA, and the fluorescence intensity of Cy5 inside cells was detected by flow cytometry.

#### Immunofluorescence

Thyroid tissues were dissected from the neck of BALB/c mice after euthanasia and immediately placed at −80 °C for the preparation of frozen sections. Frozen sections were divided into three groups. The XI group was incubated with Cy5-XI solution, the TDH group was incubated with Cy5-TDH, and the positive group was incubated with anti-Tg antibody. DAPI was used to stain the nucleus, and the slides were sealed after being washed three times. The staining of the sections was observed under a fluorescence microscope, and the fluorescence images were obtained by scanning.

#### DNA–Protein Interaction Experiment

TPC1 cells were seeded in 10 cm dishes and incubated for 6 h with 100 μL of M-TDH or M-DH with the same Mn^2+^ content. The whole protein of TPC1 was extracted, and Tg protein was pulled down using Tg monoclonal antibody and protein A/G magnetic beads. Nucleic acids co-precipitated with proteins were extracted using the BeyoChIP assay kit. The specific binding of Tg protein to the nucleic acid sequence of the Tg aptamer in M-TDH was detected by RT-qPCR using specific primers.

#### In Vitro Cytotoxicity Assay

The cell viability of TPC1, K1, Nthy ori-3.1, MDA-MB-231, MCF-7, and MCF-10A cells was determined by CCK-8 assay. Cells were seeded into 96-well plates at a density of 1000 cells per well and cultured in 5% CO_2_ at 37 °C overnight. The cells were incubated with M-TDH, TDH, and MnCl_2_ at different concentrations for 24 h. To ensure the Mn^2+^ concentrations in MnCl_2_ and M-TDH was consistent, 20 μL of 0, 0.1, 1, 2, 4, 8, and 10 mM concentrations of MnCl_2_ were added to 1 mL culture medium for cell incubation. Similarly, 20 μL of M-TDH and corresponding TDH with concentrations of 0, 17.9, 179.4, 358.9, 717.8, 1435.5, and 1794.4 ng/μL were added into 1 mL of culture medium for cell incubation. After rinsing with PBS 3 times, the cells were incubated with FBS-free RPIM-1640 (100 μL/well) supplemented with 10% CCK-8 reagent for 2 h. The absorbance at 450 nm was measured by a microplate analyzer. For each sample, five parallel wells were treated and analyzed to calculate the mean value and standard deviation of cell viability.

#### Cell Apoptosis Assay

As for apoptosis flow tests, the cells were seeded into six-well plates at a density of 5 × 10^5^ cells per well. After incubating with 20 μL of 897.2 ng/μL M-TDH and TDH, or 5 mM MnCl_2_ in 1 mL culture medium for 24 h (Mn^2+^ concentration in both M-TDH and MnCl_2_ group was 5 mM), the cells were trypsinized (without EDTA) and centrifuged at 1000 rpm for 5 min. The collected cell precipitate was washed with PBS three times, resuspended in Annexin V binding buffer (100 μL), then incubated with 5 μL of Annexin V-FITC at room temperature for 10 min in the dark, and stained with 5 μL of PE for another 10 min. The cell apoptosis rates were measured by flow cytometry. The different labeling patterns in the Annexin V-FITC/PE analysis were used to identify the different cell populations. The data analysis was performed using FlowJo software. As for the live/dead cell staining assays, 8000 TPC1 or K1 cells were cultured on 96-well plates for 24 h and were categorized into distinct treatment groups (the same as above). After washing twice with PBS, 100 μL of detection working solution with 1:1000 diluted calcein acetoxymethyl ester (AM) and propidium iodide (PI) was added to each well. After incubation at 37 °C for 30 min, the cells were observed and photographed under a fluorescence microscope, and Calcein AM stained live cells showed green fluorescence. The dead cells were stained with PI and showed red fluorescence.

#### Acid Promoting Mn^2+^ Release

A 50 μL amount of 6 mg/mL M-TDH was added into 450 μL of PBS with different pH values and then incubated in a shaker at room temperature. At selected time points, after 12 000 rpm and 10 min of centrifugation, 10 μL supernatant solutions were extracted to measure Mn^2+^ concentration released by ICP-MS under a mildly acidic environment, and 10 μL of PBS buffer of the same pH was added back into the same tube.

#### Hemolytic Test

A 1 mL portion of blood was added into 10 mL of saline and centrifuged at 10000*g* for 5 min, and the supernatant was removed. This process was repeated 3 times until the supernatant was clear. Red blood cells were resuspended in 10 mL of saline. An 800 μL amount of H_2_O for positive control, 800 μL of PBS for negative control, and 800 μL of normal saline containing 50 μL of M-TDH for the experimental group were added to a 200 μL red cell suspension. Hemolysis was photographed after incubation at 37 °C for 4 h. The absorbance at 577 nM was measured by 100 μL of the supernatant in a 96-well plate.

#### In Vivo Distribution Study

All animal experiments were performed according to the animal use and care regulation and the animal management rules of the Ministry of Health of the People’s Republic of China ([2022] IACUC Number: 3903). The BALB/c mice were bought from BNT (Hubei, China). BALB/c mice with an average 20 g body weight were divided into three groups (*n* = 3). After full consideration of biological safety,^21^ 50 μL of solvent containing 0.04 mmol/mL Gd-DPTA, 5 mM MnCl_2_, or 897.2 ng/μL M-TDH was injected via the tail vein, respectively. The concentration of Mn^2+^ was 5 mM in both MnCl_2_ and M-TDH. The body weights of mice were measured at day 0, day 3, and day 14. The blood biochemical indexes were quantified by the clinical laboratory of Boerfu Biotechnology Co., Ltd. Major organ slices were stained with H&E and observed under a light microscope.

#### In Vivo Imaging of M-TDH

The NCG mice were purchased from GemPharmatech (Jiangsu, China). Mice bearing both a TPC1 thyroid tumor and an MDA-MB-231 breast tumor were chosen to evaluate the T1-weighted imaging performance. The NCG mice were divided into three groups (*n* = 3) and injected with 50 μL of Gd-DPTA, MnCl_2_, or M-TDH via the tail vein, respectively. T1-weighted MR images were collected at different times using a 3.0 T magnetic resonance imaging system. The parameters adopted were as follows: TR/TE = 377/14 ms; FOV (AP (freq) × RL (phase) × FH) = 36 × 35 mm × 23 mm; Voxel (AP (freq) × RL (phase) × FH) = 0.25 × 0.25 mm × 2 mm; Matrix = 144 × 129 × 8 slices, gap = 1 mm; fat saturation SPIR. The relative T1 signal intensities of thyroid tumors and breast tumors, kidneys, and livers were analyzed to evaluate the MR imaging performance. The biodistribution of Mn in thyroid and breast tumors as well as major organs such as the kidney and liver was measured by ICP-MS.

#### Data Analysis

The experimental data were analyzed by one-way ANOVA. The analysis was conducted in Graphpad Prism 9.0 software. A *p* value of <0.05 is chosen as the significance level; all data are marked as * (*p* < 0.05), ** (*p* < 0.01), *** (*p* < 0.005), and **** (*p* < 0.0001).

## 3 Results and Discussion

### 3.1 Construction and Characteristic of Tg-Targeted and pH-Responsive Biomineralization DNA Hydrogel

**Figure 1:**
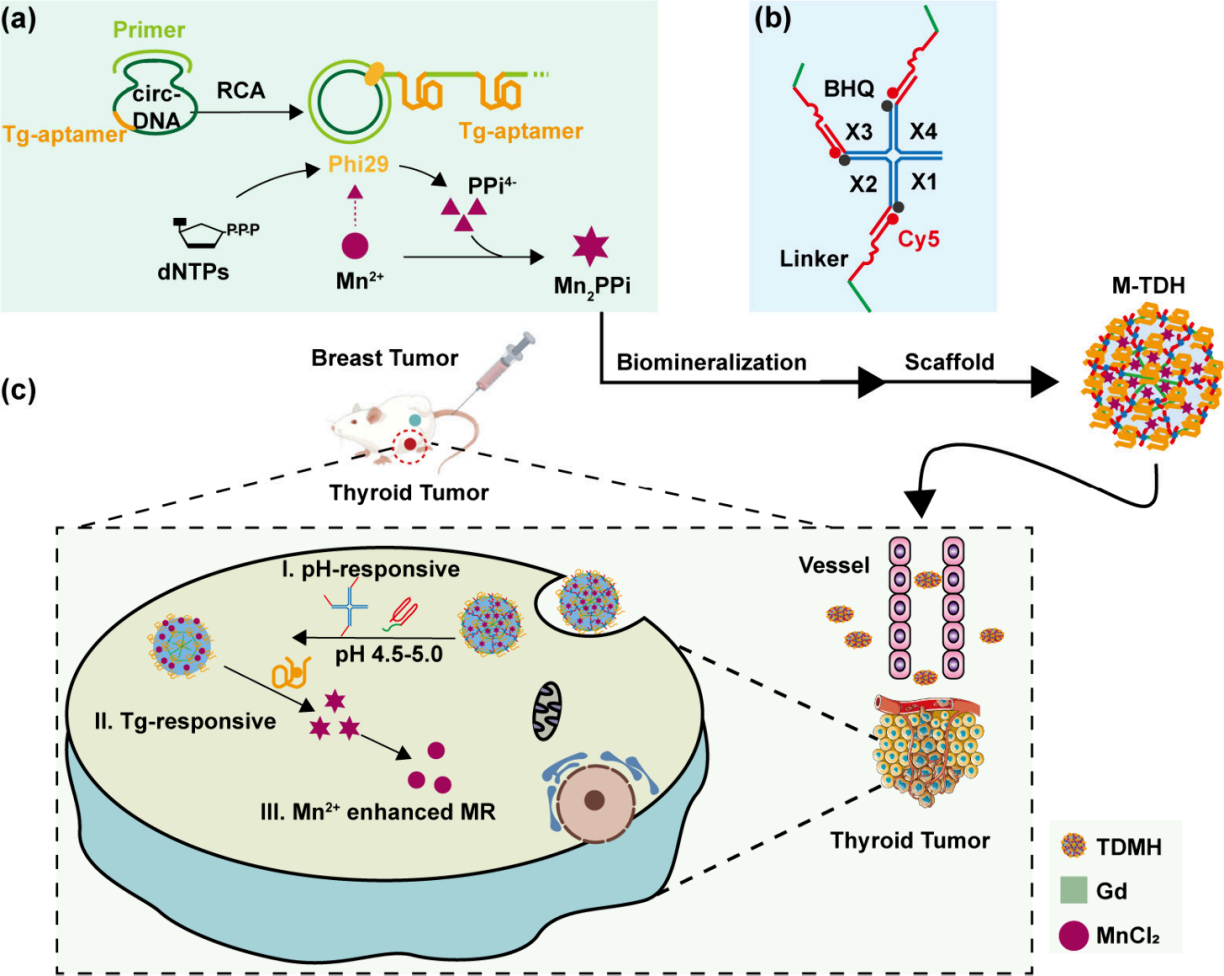
The synthesis and principal of M-TDH. (a) The biomineralization process. (b) The structure of the X-scaffold labeled BHQ1 and I-linker labeled Cy5. (c) M-TDH targeting thyroid tumor, pH- and Tg-responsive destruction, and release of Mn^2+^ to facilitate T1-weighted MR enhanced imaging.

As illustrated in Figure 1(a), paramagnetic Mn^2+^ serves as a cofactor for the φ29 DNA polymerase, facilitating the extension of long single-strand DNA (ssDNA) during the RCA reaction. The RCA process generates a considerable amount of PPi^4–^ in solution, where free Mn^2+^ ions interact with PPi^4–^ to form insoluble Mn_2_PPi, which serves as the mineralized skeleton for nanoflowers, while the long ssDNA acts as the biological template for mineralization. Using a biomineralized DNA nanogel as a pH-sensitive MRI CA, a noninvasive imaging method targeting thyroid-derived tumors for enhanced MRI is proposed. To achieve this, the complementary sequence of Tg aptamer (Table S1) was integrated into a circular template, and Mn^2+^ ions were introduced in the form of MnCl_2_ solution into the RCA reaction system.^22^ Ultimately, a DNA nanohydrogel targeting Tg was synthesized, named the Tg-targeted DNA hydrogel (TDH). When Mn^2+^ was incorporated, it was designated as Mn-loaded TDH (M-TDH).

To enhance the stability and pH responsiveness of M-TDH, the XI-scaffold was incorporated into long ssDNA (Figure 1(b)). The XI-scaffold consists of four single strands, named X1–X4, and three I-linkers. Under acidic conditions, the I-linker folds into a triplex-DNA structure, leading to disintegration of M-TDH, thereby endowing M-TDH with pH-responsive capabilities. M-TDH is expected to maintain its nanostructure stably in extracellular physiological environments and disassemble upon entering the acidic tumor microenvironment (TME, pH < 6.8, thereby releasing Mn^2+^ to enhance MR imaging (Figure 1(c)).

**Figure 2:**
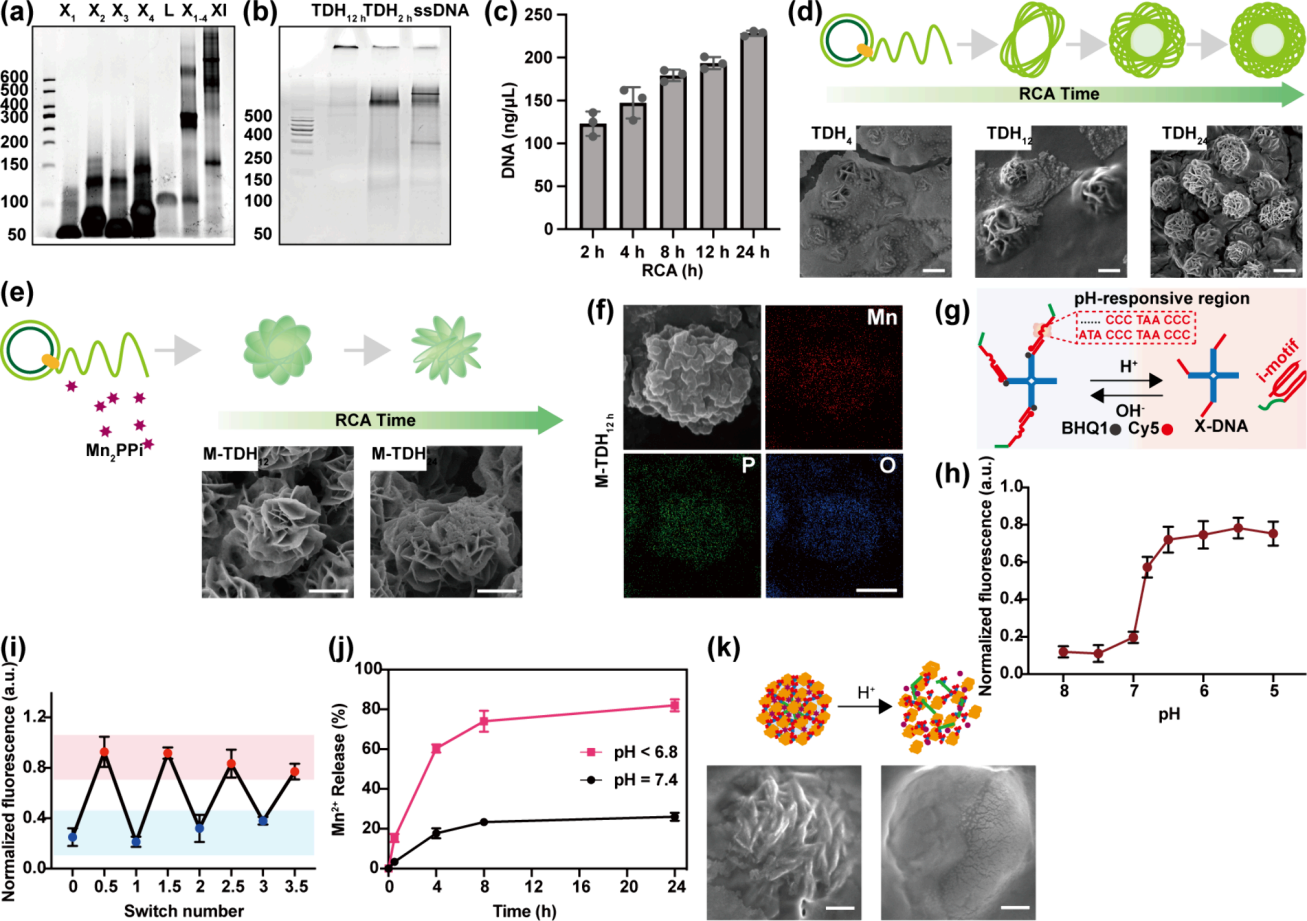
Characterization of M-TDH. (a) The formation of an XI structure shown by 2.5% agarose gel electrophoresis. (b) TDH shown by 12.5% native-PAGE electrophoresis. (c) DNA concentration in the RCA reaction mixture. (d) Schematic illustration of the time-dependent evolution of TDH and SEM images of TDH with specific RCA time (scale bar 1 μm). (e) Illustration of the formation of M-TDH with 8 mM Mn^2+^ added and SEM images of M-TDH with a specific time (scale bar 200 nm). (f) Elemental spectroscopy analysis showed a uniform distribution of Mn, O, and P within M-TDH. (g) Schematic of the pH-switched XI structure. (h) Fluorescence intensity analysis of the XI structure under difference pH conditions (*n* = 3). (i) Switch cycles of the XI structure between acidic and neutral pH. The normalized fluorescence of Cy5 at 663 nm was recorded as the readout signal (*n* = 3). (j) Mn release rate under physiological or acidic conditions detected by ICP-MS. (k) Scanning electron microscopy image of M-TDH under pH 7.4 and acidic pH < 6.8 (scale bar 100 nm).

The formation of the XI-scaffold was characterized using 2.5% agarose gel electrophoresis (Figure 2(a)). The short strands X1–X4 or the linker alone moved rapidly and corresponded to the expected molecular weight (lanes 1–5). Lane 6 showed the product of four complementary X-strands. The XI scaffold (lane 7) exhibited a larger band, indicating successful base pairing and assembly of the X-scaffold with I-linker. The generated TDH (RCA reaction and biomineralization product) was confirmed by 12.5% PAGE electrophoresis analysis. TDH (lanes 1, 2) was retained in the well of the gel, migrating much slower than linear ssDNA (lane 3) due to its higher molecular weight after the RCA reaction. Electrophoresis mobility was hindered with the extension of the RCA reaction time (Figure 2(b)). The time-dependent increase of the concentration of long ssDNA during the RCA process was evaluated using a Nanodrop spectrophotometer. The RCA mixture was pretreated with EDTA to disrupt the M-TDH nanostructure, and the DNA concentration was quantified by measuring the characteristic absorbance at 260 nm. As the reaction time was extended, the DNA concentration in the RCA reaction mixture gradually increased from 123 to 228 ng/μL (Figure 2(c)). The biomineralization process relies on the generation of long ssDNA. The morphology of long ssDNA products was first compared by SEM after 4 h, 12 h, and 24 h RCA reactions. The TDH generated by the *n*-hour RCA reaction was denoted as TDH_*n*_. Scanning electron microscopy (SEM) images revealed that TDH had a multilayer network structure consisting of intersecting nanosheets. As the reaction time was extended from 4 h to 24 h, the diameter remained unchanged (1.308 ± 0.335 μm), but the network structure became denser (Figure 2(d)). The thickness of the intersecting nanosheets was 0.095 ± 0.0387 μm. Since the reticular structure of TDH_4_ was too loose, products from reactions longer than 12 h were selected for further study. In the mineralization reaction, the free dimethylmetal cation reacted with the phosphate group (P_2_O_4_^4–^) generated by the RCA reaction to spontaneously and continuously form the floral nanostructure with the long ssDNA.^23^ It has been reported that for the preparation of Mn-based DNA nanomaterials, 8 mM Mn^2+^ is required to obtain a bouquet with a diameter of 1.5 μm.^18^ Therefore, 8 mM Mn^2+^ was added to TDH_12_ and TDH_24_. The number of nucleation sites in Mn_2_PPi nanocrystals increased, which helped avoid the formation of larger crystal structures. SEM images (Figure 2(e)) revealed that the individual rosette structure size of M-TDH_12_ was 426.4 nm, while the size of M-TDH_24_ increased to 693.8 nm. Dynamic light scattering (DLS) confirmed that the hydrodynamic average size of the M-TDH_12_ particle was 393.3 ± 15.2 nm (Figure S1), which is suitable for further biological application. It has been reported that nanoparticles with a diameter ranging from 50 to 500 nm can be taken up by endocytosis. However, when the particle size exceeds 500 nm, nanoparticles are taken up by phagocytosis.^24^ The nanoparticles selected for this study were all smaller than 500 nm, suggesting that they can be endocytosed by the cells.

Elemental spectroscopy analysis was then used to investigate the elemental distribution within M-TDH. Representative elements of the mineralized organic template DNA and inorganic component Mn_2_PPi, namely, oxygen (O), phosphorus (P), and manganese (Mn), were uniformly distributed within the porous structures (Figure 2(f)). The composition of M-TDH was further characterized using X-ray photoelectron spectroscopy (XPS). The XPS spectral analysis showed peaks for constituent elements, including O_1s_ (531.21 eV), C_1s_ (284.21 eV), P_2p_ (133.23 eV), and Mn_2p_ (641.32 eV) (Figure S2). Semiquantitative analysis based on XPS data revealed that the weight percent of DNA in M-TDH was approximately 19.3%.

The nanoparticle hydrogel exhibited excellent pH-responsive capabilities. The DNA triplex structure facilitated the release of Mn^2+^ in the acidic TME and lysosome, thereby improving MRI performance at the tumor site. The Cy5-labeled I-linker was mixed with the BHQ1-labeled X-scaffold. In the XI structure, the Cy5 fluorescence was quenched by BHQ1 under an alkaline environment. Under acidic conditions, the I-linker dissociated from the X-scaffold as the triplex-DNA structure folds, leading to Cy5 fluorescence emission. The fluorescence intensity of X_BHQ1_+I_Cy5_ was tested under different pH conditions (Figure 2(g) and 2(h)). As the pH decreased, the Cy5 fluorescence gradually recovered. By adjusting the pH, fluorescence quenching and recovery of X_BHQ1_+I_Cy5_ can be repeatedly switched (Figure 2(i)). Next, the Mn^2+^ release was detected by ICP-MS. M-TDH demonstrated good stability under physiological conditions and rapid Mn^2+^ release under acidic conditions (Figure 2(j)). The appearance of M-TDH is shown in visual images (Figure S3), and its nanostructure under different pH conditions was examined using SEM (Figure 2(k)). M-TDH displayed a well-defined mesh-like cross-linked structure at neutral conditions (pH = 7.4) and disintegrated and collapsed in acidic buffer (pH < 6.8).

The Tg aptamer encoded within the long ssDNA imparts M-TDH with the ability to specifically recognize Tg, enabling its targeted binding to thyroid cancer. The Tg aptamer was replaced with poly-A within the circ-DNA template to generate the Mn-loaded DNA hydrogel (M-DH) without Tg targeting ability. M-TDH and M-DH were used as templates for a PCR reaction, with primers complementary to the Tg aptamer designed to confirm the presence of the Tg aptamer characteristic sequence in M-TDH, while M-DH failed to produce the corresponding product. The cycle threshold (CT) values of the PCR reaction demonstrated the presence of the Tg aptamer template in M-TDH but not in M-DH (Figure S4).

### 3.2 In Vitro Stability and Cytotoxicity

**Figure 3:**
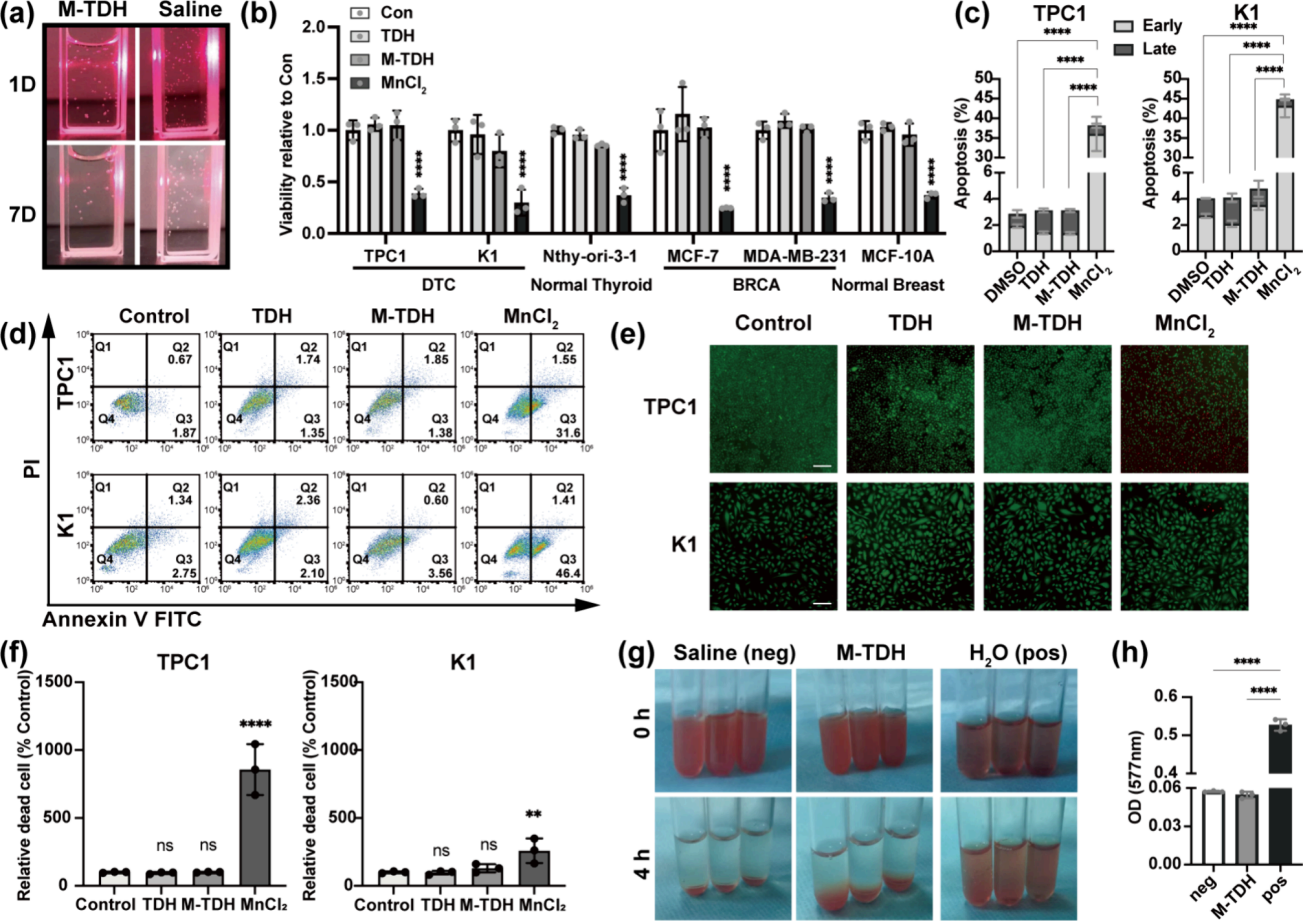
In vitro stability and cytotoxicity of M-TDH. (a) Tyndall effect showed good stability of M-TDH in saline with 10% FBS. (b) Cell viability after 24 h of incubation with PBS, TDH, M-TDH, and MnCl_2_, detected by CCK-8. (c, d) Cell apoptosis rates of DTC cells detected by flow cytometry. (e, f) Live/dead cell staining images and corresponding quantitative comparison of TPC1 and K1 cells after various treatments (scale bar 100 μm). (g, h) Hematological parameters of M-TDH detected by hemolysis experiments and corresponding quantitative measurements.

M-TDH exhibits excellent colloidal stability in aqueous solutions. At room temperature, M-TDH remained evenly dispersed in PBS containing 10% serum. After 1 week, no aggregation was detected, and a clear Tyndall effect was observed (Figure 3(a)). The toxic effects of the nanohydrogel containing various concentrations of Mn^2+^ on thyroid cancer cell lines (TPC1 and K1), breast cancer cell lines (MCF-7 and MDA-MB-231), and normal cell lines (Nthy-ori-3-1 and MCF-10A) were investigated. Cell survival rates after 24 h of incubation with M-TDH or TDH were measured using the CCK-8 (Figure S5). Both M-TDH and TDH demonstrated high cell viability across a broad range of Mn^2+^ concentrations. However, incubation with MnCl_2_ for the same duration reduced cell viability to approximately 30% (Figure 3(b)). The apoptosis rates of thyroid cancer cells, detected by flow cytometry, showed results similar to those from CCK-8 assays (Figure 3(c) and 3(d)). To visually assess the vitality of living and dead cells, we costained cells with AM and PI solution. This costaining allows for the distinction of live cells emitting green fluorescence from dead cells emitting red fluorescence. The control, TDH, and M-TDH groups exhibited minimal or no cell damage, while a majority of dead cells were evident in the MnCl_2_ group (Figure 3(e) and 3(f)).

Hematological parameters of M-TDH were also assessed by measuring the absorbance after incubation with a red blood cell suspension (Figure 3(g) and 3(h)). Minimal hemolysis (less than 0.1) indicated that M-TDH is suitable for in vivo applications, particularly for intravenous administration.

### 3.3 Cellular Uptake and Tg Targeting

**Figure 4:**
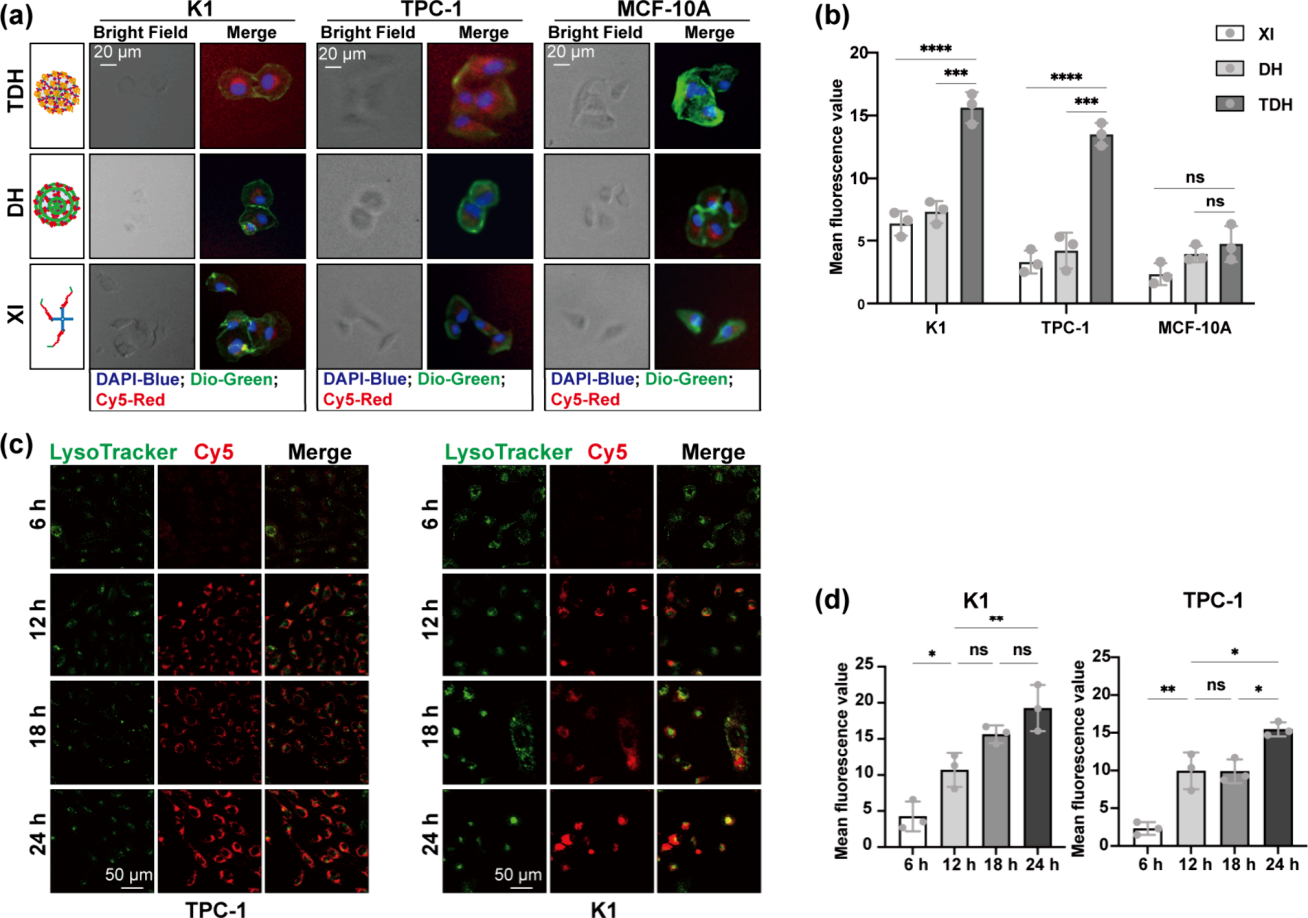
Cellular uptake and thyroid tumor-targeting ability of the nanohydrogel. (a, b) Red signal and mean fluorescence value in K1, TPC1, or MCF-10A after 12 h of incubation with XI, DH, or TDH, detected by CLSM. (c, d) Red signal and mean fluorescence value in K1 and TPC1 with prolonged incubation time detected by CLSM.

The thyroid tumor-targeting properties of TDH were evaluated using Tg positive thyroid cancer cells (TPC1 and K1) and negative control cells (human breast epithelial cells, MCF-10A). The Cy5-labeled XI structure was incorporated into the assembly of TDH, forming Cy5-TDH (red fluorescence). Naked Cy5-XI, nonaptamer Cy5-DH, and Cy5-TDH were incubated with three cells. The uptake of Cy5-TDH by thyroid cancer cells was indicated by the emission of red fluorescence. The distribution of nanohydrogels within cells was visualized by using CLSM. After 12 h of incubation, thyroid cancer cells exhibited brighter red fluorescence compared to the negligible red fluorescence in MCF-10A, suggesting that TDH possesses thyroid tumor-targeting ability, which depends on the Tg aptamer (Figure 4(a) and 4(b)).

The cellular uptake of the nanohydrogel was further investigated in thyroid cancer cells. The red fluorescence signal was used to indicate cellular uptake after 6, 12, 18, and 24 h of incubation. The endocytic pathways of M-TDH entering thyroid cancer cells were studied by selectively staining lysosomes with LysoTracker Green. CLSM results showed a faint red fluorescence signal in the cells after 6 h of incubation; after 12 h of incubation, higher colocalization of red and green signals was observed, with an increased red fluorescence signal within the cells, which then dispersed throughout the cells (Figure 4(c) and 4(d)).

**Figure 5:**
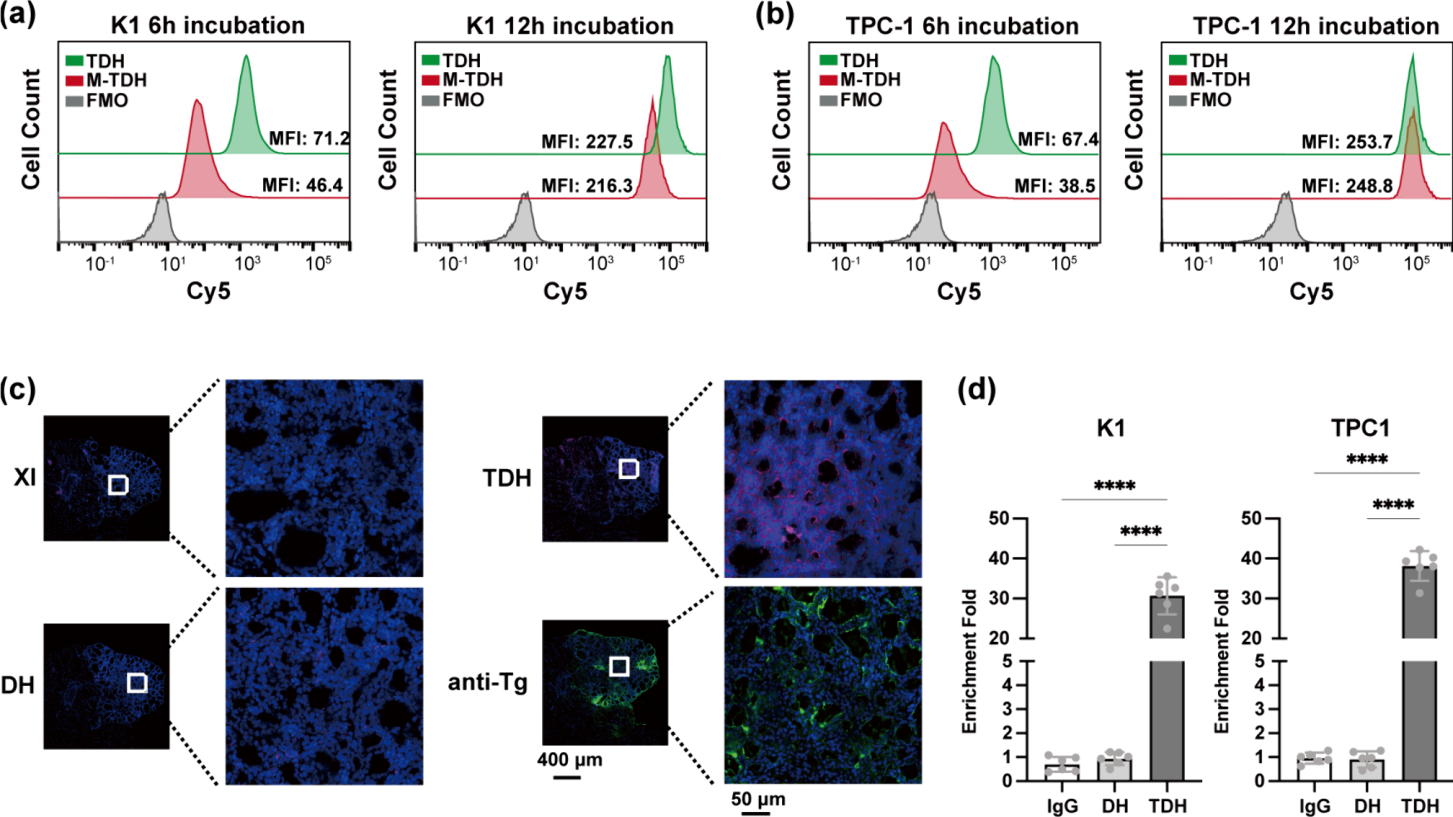
Uptake efficiency and aptamer-mediated combination. (a, b) The uptake efficiency of M-TDH or TDH by K1 and TPC-1 cells detected by flow cytometry. (c) The in situ targeting of the nanohydrogel in frozen thyroid tissue sections. The white box shows the enlarged area, Cy5-TDH shows a red fluorescence signal, and a green fluorescence signal indicates the Tg distribution. (d) Nucleic acid–protein binding assay used to compare the combination between DH or TDH and endogenous Tg protein in the cellular environment.

Flow cytometry was used to compare the uptake efficiency of M-TDH and TDH in thyroid cancer cells. At 6 h of incubation, M-TDH uptake was lower than that of TDH, probably due to the larger size of M-TDH. However, after extending the incubation time beyond 12 h, M-TDH was completely taken up by the cells (Figure 5(a) and 5(b)), indicating that the presence or absence of Mn^2+^ did not affect the cellular uptake of the nanohydrogel.

To investigate the in situ targeting of TDH in tissues, frozen thyroid tissue sections were used (Figure 5(c)). The results showed that the specificity of intact TDH was similar to that of the primary Tg antibody. To further confirm that the targeting of TDH to thyroid tissue/cells results from specific binding to Tg, a nucleic acid–protein binding assay was conducted (Figure 5(d)). The results revealed that sequences specific to TDH were detected in the nucleic acids pulled down by the Tg antibody, and BLAST analysis confirmed that these sequences were absent in the human DNA sequence.

### 3.4 In Vivo Pharmacokinetics and Biodistribution

**Figure 6:**
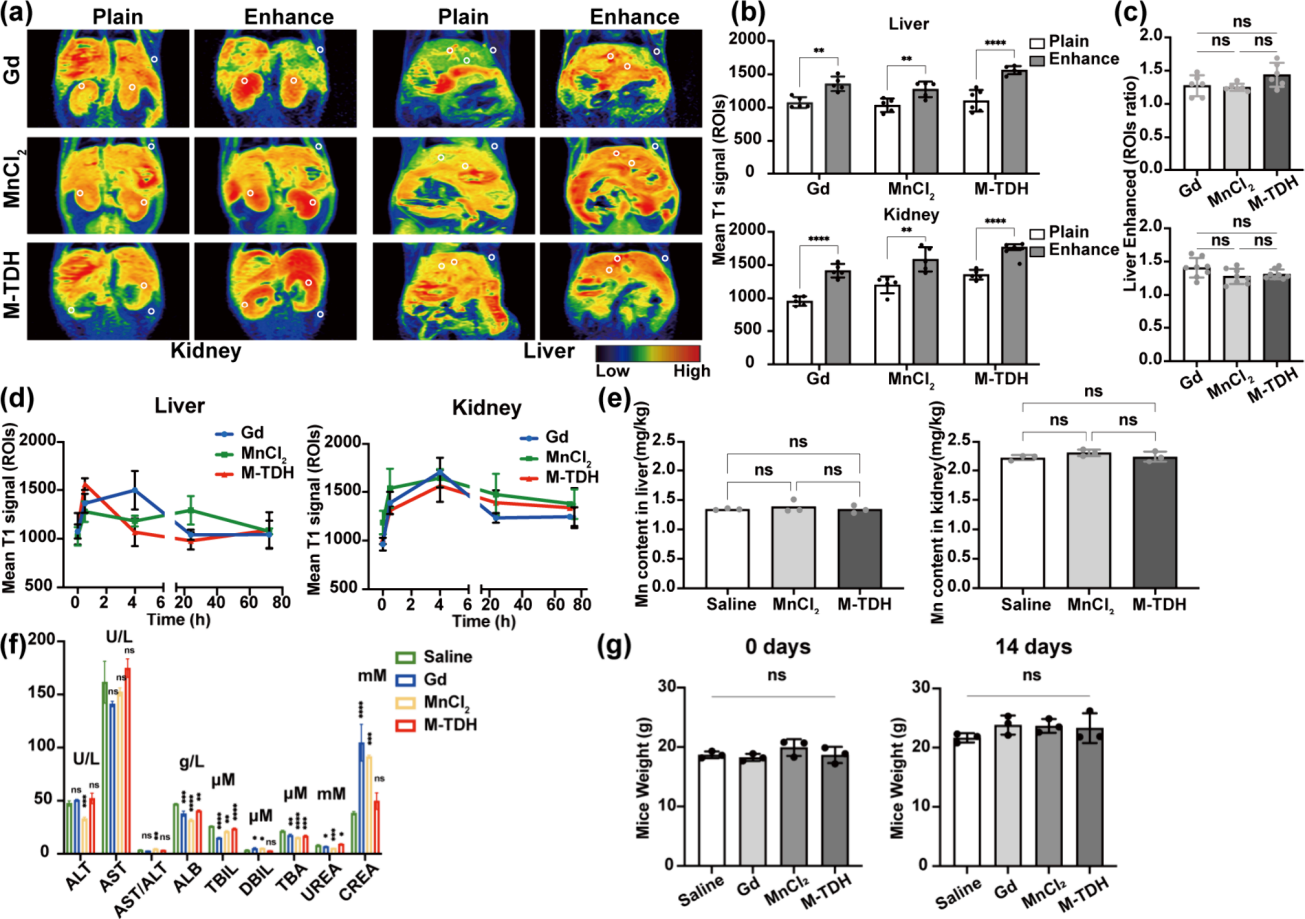
In vivo MR enhancement and biodistribution of M-TDH, MnCl_2_, and Gd-DPTA. (a) Plain and enhanced MR images of liver and kidney. White circles show the ROI measurement sites; the left and right kidneys, liver vessels, parenchyma, and adjacent muscles as baseline signal were measured. (b) Mean ROIs of plain and enhanced MR show the T1MR signal on the liver and kidney. (c) Enhancement of the ROI ratios shows enhanced T1 intensity of Gd, MnCl_2_, or M-TDH. (d) MR signal intensity decreased with time and nearly returned to the initial level after 72 h. (e) Mn content in the liver and kidney was detected by ICP-MS. (f) Liver and kidney function in every group were detected and compared by blood chemistry analysis. (g) Mice weight before and 14 days after injection in every group.

M-TDH, MnCl_2_, and Gd-DPTA (Gd) were administered via tail vein injection into BALB/c mice. MRI of the liver and kidneys was performed before injection, immediately after injection, and at 4, 24, and 72 h postinjection. The enhancement effects of the CAs in vivo were assessed through immediate imaging (Figure 6(a)–(c)), while specific time-point MR imaging monitored the metabolic processes of CAs in the body (Figure S6). The liver’s immediate signal significantly increased after injection, whereas the kidney’s MR signal peaked at 4 h postinjection. Continuous imaging of the liver and kidneys revealed a noticeable decrease in the MR signal intensity at 24 h. At 72 h postinjection, the imaging intensity in these regions had returned to the preinjection levels (Figure 6(d)), indicating effective clearance of M-TDH in vivo and providing a basis for potential safe clinical applications. ICP-MS analysis of Mn content in the liver and kidney showed no difference between the M-TDH injection group and the control group (Figure 6(e)).

After 14 days, blood chemistry analysis and histological examination were performed to assess the toxicity of M-TDH. Blood chemistry analysis of liver and kidney function showed no significant difference between the treatment group and the control group, indicating that M-TDH did not cause liver or kidney damage or malfunction (Figure 6(f)). No significant changes in body weight were observed between the saline and M-TDH groups, indicating that M-TDH did not affect mouse growth (Figure 6(g)). Gross examination and H&E staining of major organs revealed no significant abnormalities or inflammation in either the treatment or control groups (Figure S7). These results suggested that M-TDH exhibited good in vivo and in vitro biocompatibility and can be further explored for internal applications.

### 3.5 T1-Weighted MR Imaging of Thyroid Cancer

**Figure 7:**
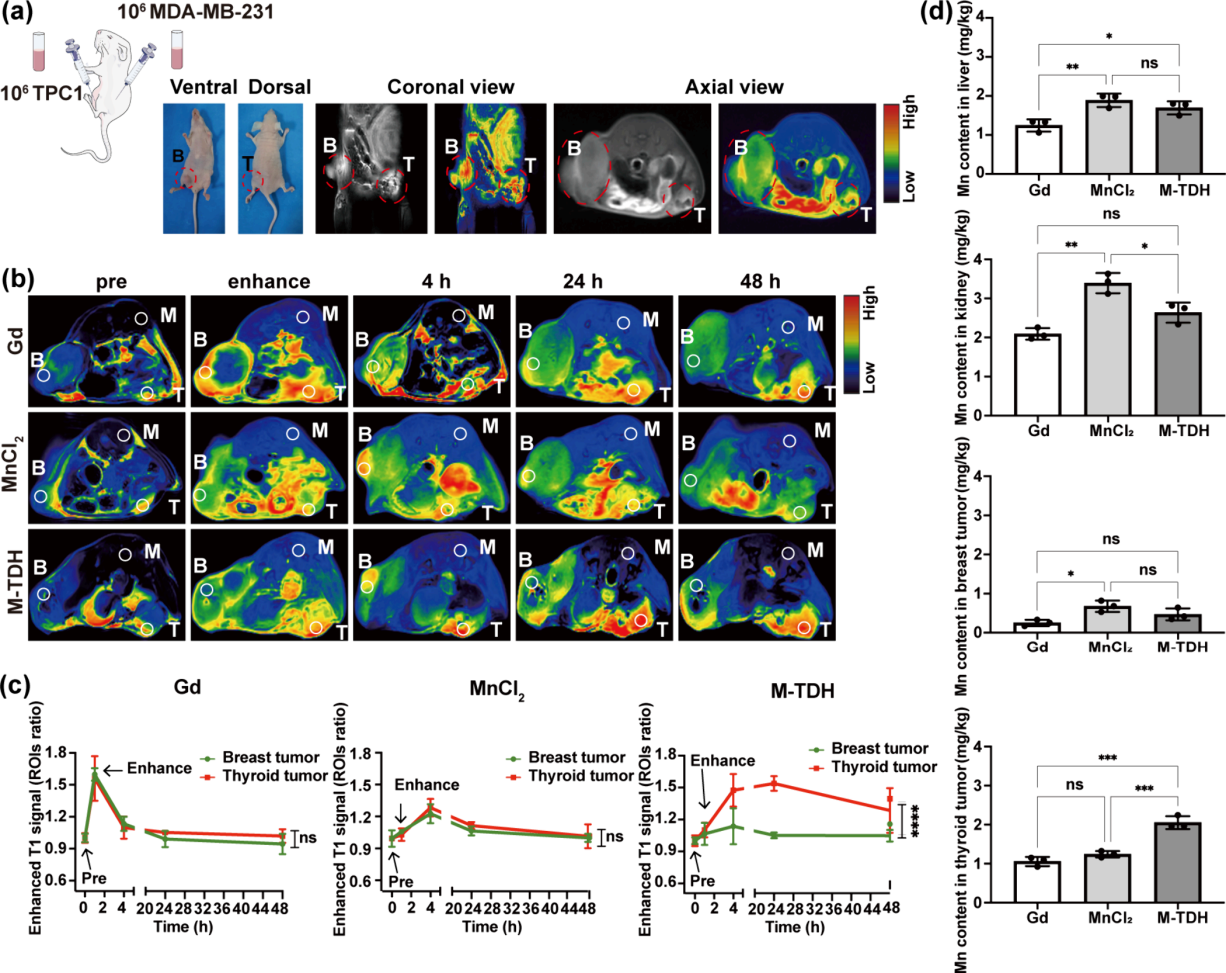
T1-weighted MR imaging of thyroid and breast tumor. (a) Tumor xenograft model construction diagram and MRI confirmation. Red circle indicates tumor region, B for breast tumor, T for thyroid tumor, M for adjacent muscles as baseline. (b) MR images and T1 intensity were detected to compare three CAs’ MR enhancement performance on thyroid or breast tumor. Specifically, preinjection, enhanced signal, and specific time (4, 24, 48 h) after injection. B indicates breast tumor, T indicates thyroid tumor, M indicates adjacent muscles as baseline signal, and white circles show ROI measurement sites. (c) Enhanced T1 signal calculated by ROI ratio (after to before) and presented by a line graph. (d) The Mn^2+^ content in tumor tissues was determined using ICP-MS after 24 h of CA injection.

An in vivo tumor model was used to evaluate the T1-MRI performance and thyroid tumor-specific targeting effect of M-TDH nanoparticles. TPC1 cells were subcutaneously implanted in immunodeficient NCG mice to establish thyroid tumor xenografts on the abdominal side, while human breast cancer cells (MDA-MB-231) were used to create breast tumor xenografts on the dorsal side (Figure 7(a)). The bi-tumor-bearing NCG mice were randomly divided into three groups and intravenously injected with M-TDH, Gd, and MnCl_2_, each presenting three different types of CAs. Continuous image acquisition was performed using the MRI system to assess the tumor imaging performance (Figure 7(b) and 7(c)). The enhancement effects on breast and thyroid tumors were analyzed at each time point.

For Gd and MnCl_2_, a significantly enhanced T1-weighted signal at the tumor site was observed immediately after intravenous injection, with the signal continuing to increase for 4 h until it reached its peak. This intense signal gradually diminished and almost disappeared within 48 h. Breast tumors, which are richer in blood vessels compared to thyroid tumors, exhibited a stronger enhanced MRI signal for both Gd and MnCl_2_. In contrast, M-TDH showed a gradual increase in signal intensity at the thyroid tumor site, peaking at 24 h. The enhanced MRI signal of M-TDH was maintained in tumor tissue for up to 48 h, significantly longer than the MRI enhancement time provided by Gd, suggesting that M-TDH-based MRI has promising applications for in vivo disease tracking.

Twenty-four hours postinjection of CAs, the Mn^2+^ content in tumor tissues and major organs was determined using ICP-MS (Figure 7(d)). Mn^2+^ ions were more concentrated in the thyroid tumors 24 h after M-TDH injection, whereas in the case of MnCl_2_ injection, the Mn^2+^ content was higher in breast tumors than in thyroid tissue and was significantly elevated in the liver and kidney. These results illustrated the tissue selectivity of M-TDH that enhanced the local accumulation of Mn^2+^ in thyroid tumors while reducing Mn^2+^ accumulation in major organs such as the liver and kidney at the same time.

## 4 Conclusion

We have developed a novel MRI CA, specifically targeting thyroid cancer and based on a DNA hydrogel, termed M-TDH. It is demonstrated that M-TDH efficiently loads Mn^2+^ through a biomineralization reaction and possesses the desired Tg targeting ability by incorporating the complementary sequence of the Tg aptamer in the RCA template. The inclusion of the X-scaffold enhances the stability of M-TDH and enables it to degrade in the acidic TME and cell lysosomes. This system is designed to selectively visualize thyroid cancer via MRI after systemic administration, aiding in the post-thyroidectomy assessment of thyroid cancer patients.

MR is a preferable method to identify concealed or uncertain thyroid tumor. Gadolinium-based contrast agents (GBCA) are the most commonly used MRI CA in clinical practice. However, in patients with renal impairment, GBCA can cause nephrogenic systemic fibrosis and its effect on individuals with normal renal function remains unclear.^25^ Additionally, small molecular CAs often exhibit relatively low relaxation rates and short circulation times, leading to their rapid excretion via the kidneys. More importantly, their lack of selectivity leads to nonspecific accumulation in physiological environments, thereby reducing the effective concentration at the target site. The combination of traditional contrast elements with a nanocarrier platform enhances imaging performance and prolongs lesion retention time.^26,27^ In this study, we used three types of CAs (M-TDH, MnCl_2_, and Gd-DPTA) in animal experiments. By comparing these different CAs, the thyroid-targeting ability and MRI-enhanced imaging performance of M-TDH were comprehensively assessed. MnCl_2_ is a simple source of Mn^2+^ that lacks targeting functionality. By comparison of MnCl_2_ with M-TDH, the MRI signal enhancement functions of M-TDH and Mn^2+^ were compared, verifying the targeting ability of M-TDH. Gd-DPTA is a clinically used T1-weighted MRI CA with widespread imaging enhancement capabilities in clinical applications. By comparing Gd-DPTA with M-TDH, the imaging enhancement effects of M-TDH relative to standard clinical contrast agents were evaluated. This not only facilitated the assessment of the imaging enhancement capabilities of M-TDH but also provided a reference for its potential clinical application. M-TDH holds promise as a novel MRI CA combining both targeting and imaging enhancement functions.

For patients who have undergone total thyroidectomy, lymph node recurrence or distant metastasis of thyroid cancer is the only source of Tg. Therefore, M-TDH, which is selectively and actively targeted to Tg, accumulates in the recurrence or metastasis thyroid cancer lesions, enhancing the MRI signal at the target site and facilitating the detection of cancer lesions. Additionally, its larger particle size prolongs the circulation time, resulting in stronger and more persistent signals in thyroid-originated cancers. M-TDH is expected to serve as a thyroid tumor-specific, persistent, and nonradioactive CA for detecting recurrence and metastasis in patients with thyroid cancer after total thyroidectomy. Furthermore, the targeting capability of M-TDH could be combined with chemotherapy and targeted drug delivery. By loading anticancer drugs into M-TDH, the drugs can be accurately delivered to the thyroid tumor site, thereby enhancing the local drug concentration and minimizing damage to normal tissues. Meanwhile, MRI imaging can monitor both the drug distribution in tissues and the tumor’s response to the treatment. The magnetic component of M-TDH enables it to generate a thermal effect under an external magnetic field, facilitating targeted hyperthermic ablation therapy when combined with magnetic resonance hyperthermia (MRHT).^28^ MR imaging can monitor changes in the thermal effect region in real time, enabling the precise localization and ablation of thyroid tumor tissue. Given the functional versatility of the DNA hydrogel in M-TDH,^29^ MRI-guided combined therapy based on M-TDH has the potential to integrate precise imaging and therapeutic approaches, allowing for real-time monitoring of therapeutic effects.

Notably, the pH-sensitive nanohydrogel may gradually degrade at neutral pH in the presence of moisture, posing a challenge to its long-term stability. Although we demonstrated that M-TDH remains stable in liquid for at least 1 week, which is sufficient for its in vivo use, longer storage remains a challenge. We hypothesized that the stability of M-TDH could be prolonged by freeze-drying followed by reconstitution with water;^30^ however, the feasibility of this approach was not further explored in this study. Additionally, our study has several limitations. We used fluorescently labeled nanoparticles and confocal microscopy, a widely used and reliable method,^31^ to visually demonstrate nanoparticle uptake and localization in cells. Given the multitargeting potential of DNA probes,^32,33^ expanding the targeting capability of M-TDH to other tumors or additional features of thyroid cancer will be a key focus of future research. Additionally, we conducted a preliminary exploration of the endocytic pathway of M-TDH by lysosomal staining with LysoTracker. However, transmission electron microscopy (TEM) was not employed to provide ultrastructural details of the nanoparticle uptake by cells. Besides, due to the difficulty in constructing orthotopic tumors resulting from the concealed location and scattered structure of the mouse thyroid gland, we used subcutaneous tumors to verify the targeting of M-TDH. The application of M-TDH in the detection of metastatic thyroid tumors and the distinguishing malignancy from benign thyroid nodules will be the direction of our future research.

In summary, our biocompatible M-TDH shows promise as a thyroid-originated cancer specific MRI nanoscale CA. It also holds potential for treatment through physically encapsulating chemotherapy drugs, making it suitable for MRI-guided combined therapy for thyroid cancer.

## Supporting Information Available

DNA sequences used in the synthesis of M-TDH (Table S1); primers’ sequences used in RT-qPCR to detect Tg aptamer in TDH (Table S2); the hydrodynamic diameter of M-TDH detected by DLS (Figure S1); XPS spectra analysis of M-TDH (Figure S2); morphology of M-TDH during synthesis and collapse (Figure S3); relative content of DNA with Tg aptamer characteristic sequence in M-TDH and M-DH (Figure S4); cell viability after 24 h treatment with different concentrations of TDH or M-TDH (Figure S5); MR images of liver and kidney before and 4, 24, and 72 h after injection (Figure S6); gross image and H&E staining of major organs assessed for in vivo biocompatibility of M-TDH, MnCl_2_, and Gd-DPTA (Figure S7) (PDF)
