# Supplementary material for: Manganese-Loaded pH-Responsive DNA Hydrogels Enable Tg-Guided Thyroid Tumor Targeted Magnetic Resonance Imaging
Source: ACS Appl Mater Interfaces. 2025 Feb 25;17(9):13403–14. doi: 10.1021/acsami.4c19676 (PMC11891823; doi:10.1021/acsami.4c19676)
Supplement: Supplementary file 1 — am4c19676_si_001.pdf [file am4c19676_si_001.pdf]

Supporting Information

Manganese-loaded pH responsive DNA hydrogels enable Tg-guided thyroid tumor targeted magnetic resonance imaging

Qingyi Hu [a §], Anwen Ren [a §], Ximeng Zhang [a §], Zimei Tang [a], Rong Wang [a], Dong-Yuan Wang [b], Tao Huang [a *], Jie Liu [c *], and Jie Ming [a *]

[a] Department of Breast and Thyroid Surgery, Union Hospital, Tongji Medical College, Huazhong University of Science and Technology, 430022, Wuhan, China

[b] Department of Pharmacy, Union Hospital, Tongji Medical College, Huazhong University of Science and Technology, 430022, Wuhan, China

[c] Department of Radiology, Union Hospital, Tongji Medical College, Huazhong University of Science and Technology, 430022, Wuhan, China

[*] E-mail of *Tao Huang*: huangtaowh@hust.edu.cn

[*] E-mail of *Jie Ming*: mingjiewh@hust.edu.cn

[*] E-mail of *Jie Liu*: E-mail: liu_jie0823@sina.com

[§] Qingyi Hu, Anwen Ren, and Ximeng Zhang contributed equally to this work.

1. Supplementary tables.

Table S1. DNA sequences used in the synthesis of M-TDH.

Name	Sequence (5' – 3')
Padlock Tg (PT-Tg)	P-ACGGGCGTCAGTGCTTCCGTGCTTGTGAGTCAAGTTAGCCTGGGC ATCGCCTCCCCGCTCACGCGGTAGCGAAGGCGAGGTAGCTGCA
Padlock polyA (PT-non)	P-ACGGGCGTCAGTGCTTCCGAAATGCTTGAAAAAAAAAAAAAAAAA AAAAAAAAAAAAA GTAGTTACGAAGGCGAGGTAGCTGCA
LT	AGTCACGCCCGTTGCAGCTACCT
RCA-primer	CCCGTTGCAGCTACCTCGCCTT
X1	CGAGTCGTTCGCAATACGGCTGTACGTATGGTCTCG
X2	GGGTATGGGTTAGGGCGAGACCATACGTACAGCACCGCTATTCATCGG TCG
X2-BHQ	GGGTATGGGTTAGGGCGAGACCATACGTACAGCACCGCTATTCATCGG TCG-BHQ1
X3	GGGTATGGGTTAGGGCGACCGATGAATAGCGGTCAGATCCGTACCTAC TCG
X4	GGGTATGGGTTAGGGCGAGTAGGTACGGATCTGCGTATTGCGAACGAC TCG
I-Linker	CCCTAACCCATACCCTAACCCCTTCCGAAATGCTTGTGAGTCAAGTTAG CC
Cy5-I-Linker	Cy5-CCCTAACCCATACCCTAACCCCTTCCGAAATGCTTGTGAGTCAAGT TAGCC

Sequence complementary to Tg aptamer was shown in red.

Table S2. Primers' sequences used in RT-qPCR to detect Tg aptamer in TDH.

Name	Sequence (5' – 3')
Primer 1	FP: TACCTCGCCTTCGCTA<br>RP: AGTGCTTCCGTGCTTG
Primer 2	FP: CCTTCGCTACCG<br>RP: CTTCCGTGCTTGTG
Primer 3	FP: CGCCTTCGCTAC<br>RP: TCAGTGCTTCCGTGCTTGT
Primer 4	FP: TACCTCGCCTTCGCT<br>RP: TGCTTCCGTGCTTG

2. Supplementary figures.

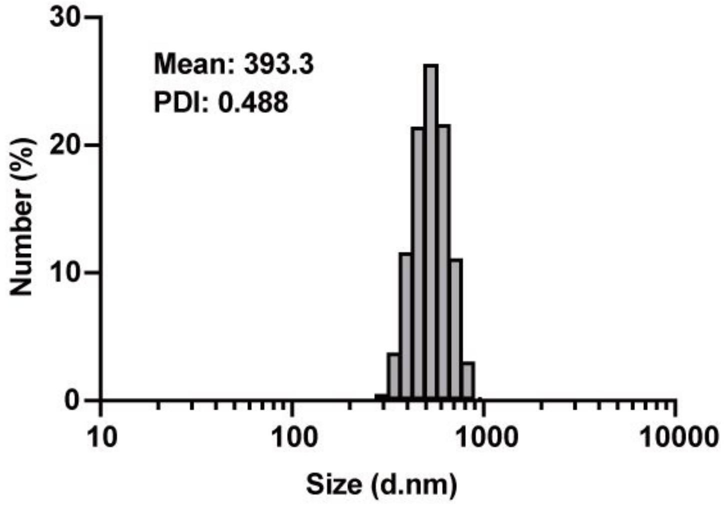

Figure S1. The hydrodynamic diameter of M-TDH.

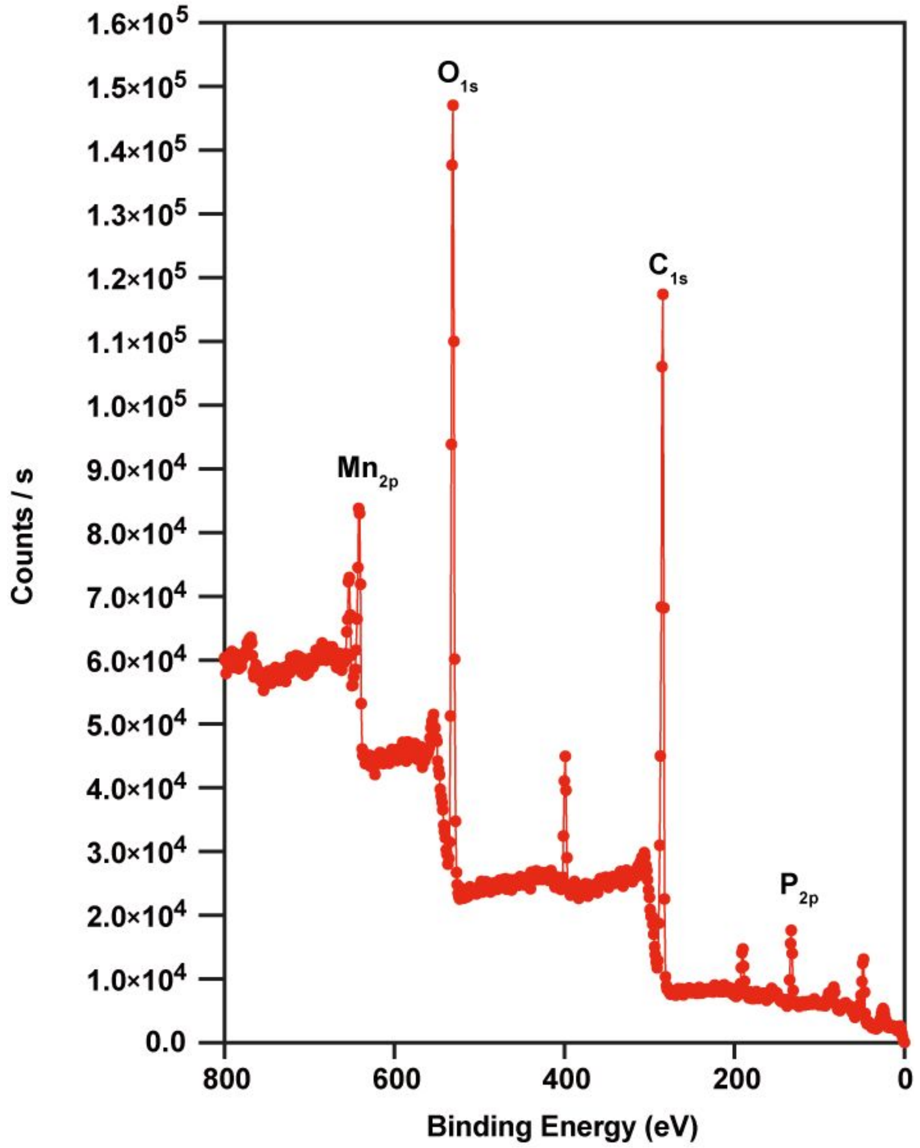

Figure S2. Survey XPS spectra analysis of M-TDH.

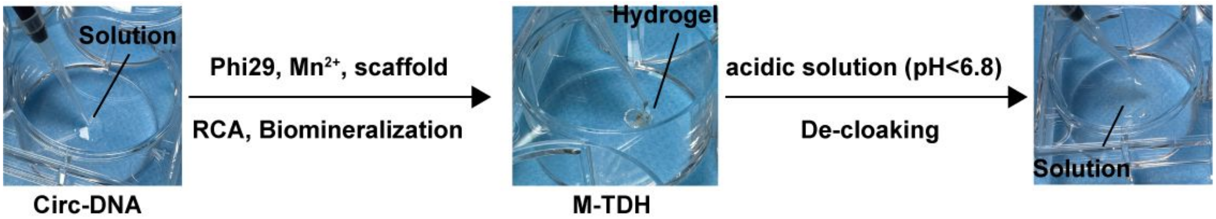

Figure S3. Visual images showed the morphology of M-TDH during synthesis and collapse.

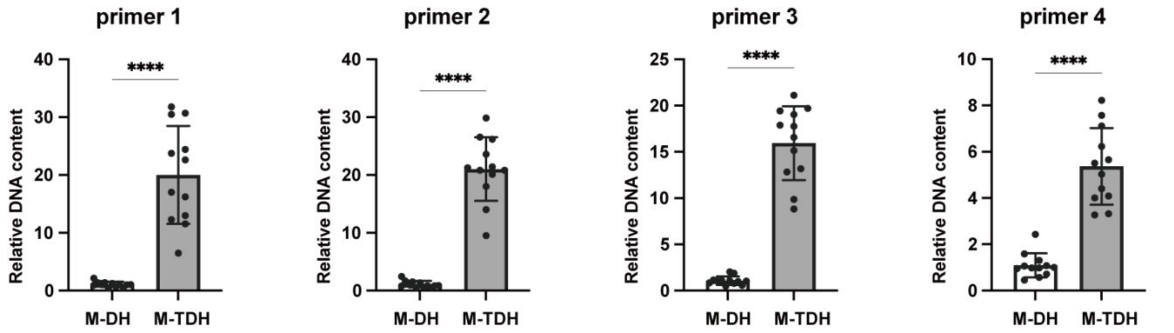

Figure S4. The PCR reaction detected relative content of DNA with Tg aptamer characteristic sequence in M-TDH and M-DH.

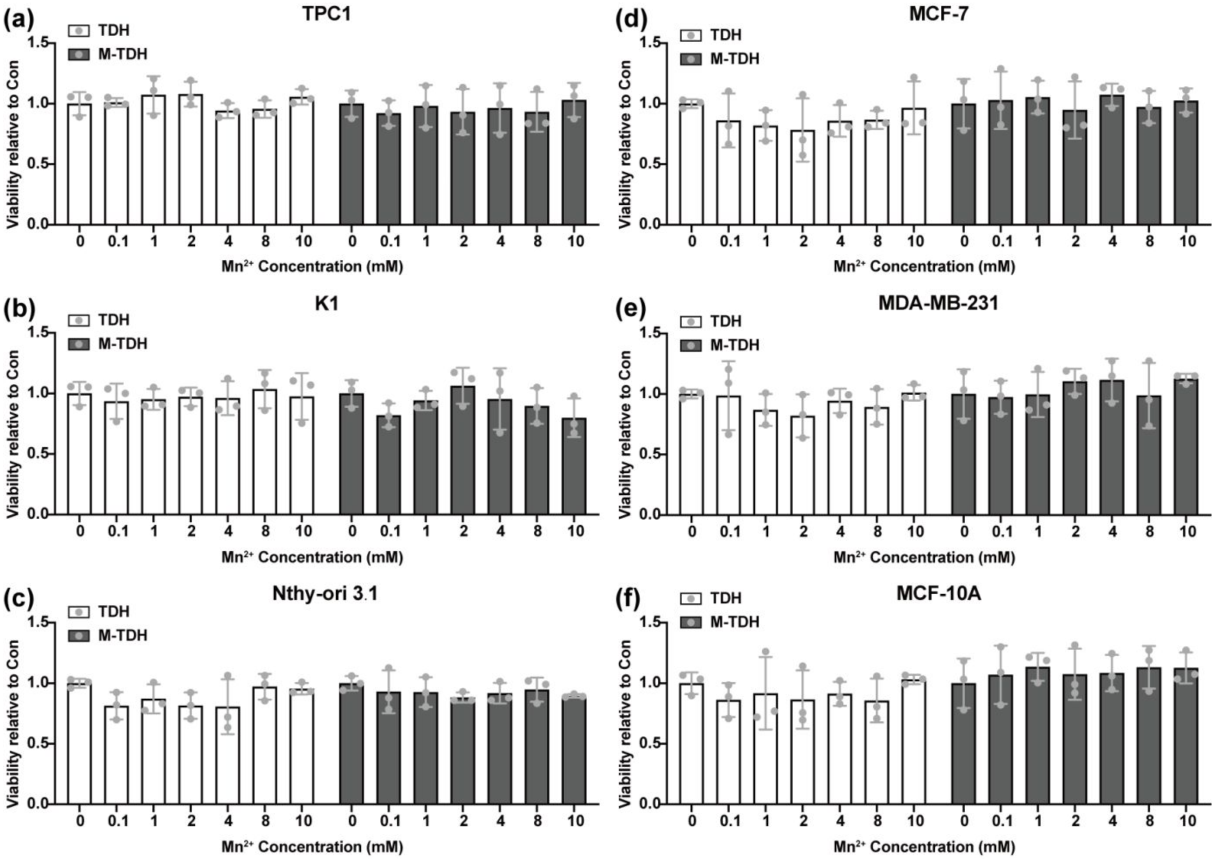

Figure S5. Cell viability after 24 h treated with different concentration of nano-hydrogel. (a-b) differential thyroid cancer (DTC) cells TPC1 and K1. (c) Normal thyroid cell, Nthy-ori 3.1. (d-e) breast cancer cells, MCF-7 and MDA-MB-231. (f) Normal breast cell, MCF-10A.

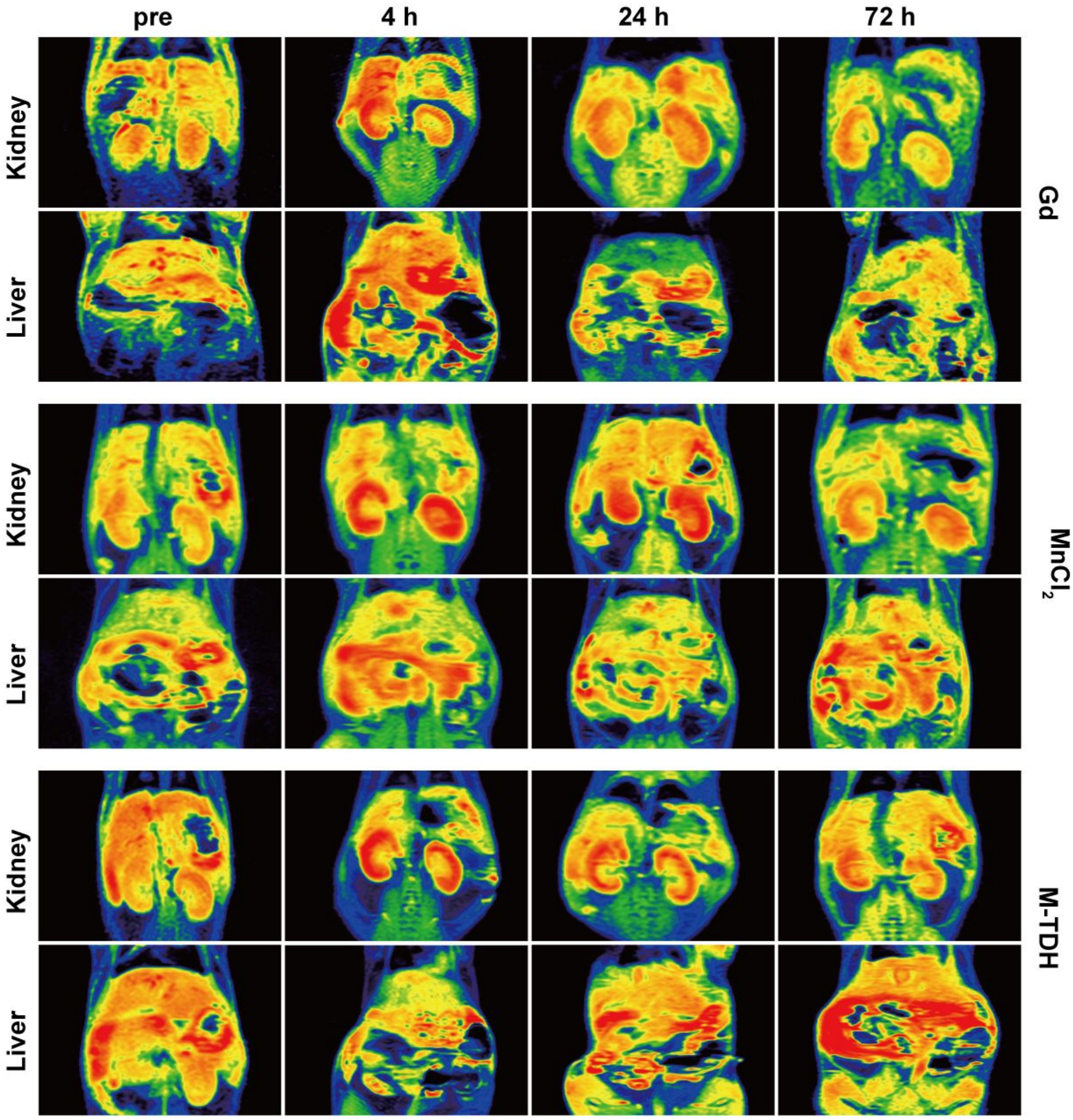

Figure S6. MR images of liver and kidney before and 4, 24, and 72 h after injection.

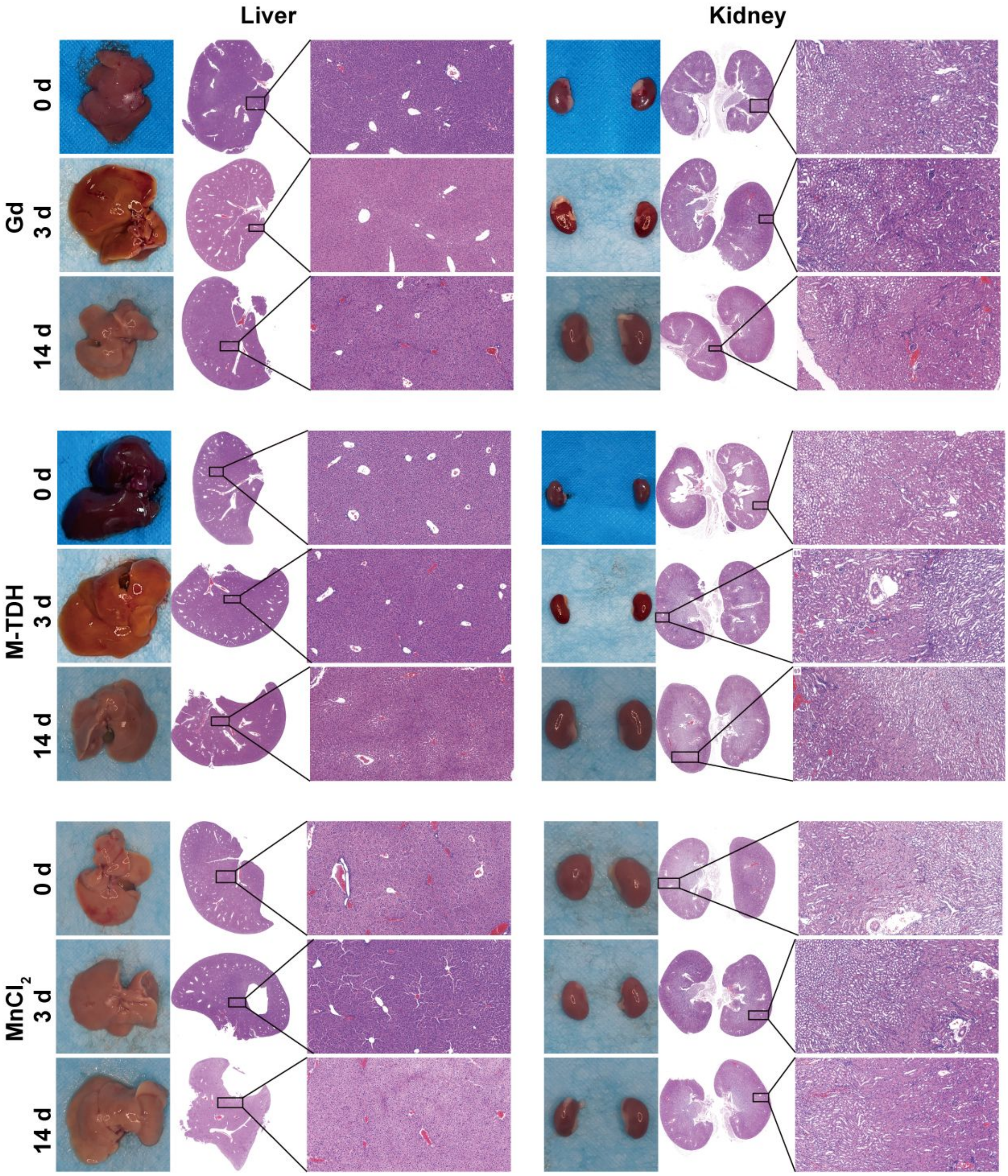

Figure S7. Gross image and H&E staining of major organs assessed after M-TDH, $MnCl_2$, and Gd-DPTA treatment.
